# Analytical validation and diagnostic performance of the *ASCL1/ZNF582* methylation test for detection of high-grade anal intraepithelial neoplasia and anal cancer

**DOI:** 10.1016/j.tvr.2023.200275

**Published:** 2023-12-30

**Authors:** Kirsten Rozemeijer, Fernando Dias Gonçalves Lima, Timo J. ter Braak, Albertus T. Hesselink, Jan M. Prins, Henry J.C. de Vries, Renske D.M. Steenbergen

**Affiliations:** a Amsterdam UMC, Location Vrije Universiteit Amsterdam, Department of Pathology, Amsterdam, the Netherlands; b Cancer Center Amsterdam, Imaging and Biomarkers, Amsterdam, the Netherlands; c Amsterdam UMC, Location AMC, Department of Dermatology, Amsterdam, the Netherlands; d Amsterdam Institute for Infection and Immunity, Amsterdam, the Netherlands; e Self-screen B.V., Amsterdam, the Netherlands; f Amsterdam UMC, Location AMC, Department of Internal Medicine, Division of Infectious Diseases, Amsterdam, the Netherlands; g Public Health Service Amsterdam, Cluster Infectious Diseases, Department of Research, Amsterdam, the Netherlands

**Keywords:** High-grade anal intraepithelial neoplasia, Anal cancer, DNA methylation, Anal biopsies, Screening, Biomarkers

## Abstract

DNA methylation testing on biopsies can detect high-grade anal intraepithelial neoplasia (HGAIN) in need of treatment and anal cancer. This study aimed to analytically validate and determine the diagnostic performance of a newly developed multiplex quantitative methylation-specific PCR, PreCursor-M AnoGYN (RUO), combining *ASCL1, ZNF582* and a reference (*ACTB*) in one assay. Analytical validation was performed on two qPCR devices using predefined quality criteria. Diagnostic performance was determined on a cross-sectional series of 111 anal biopsies covering all stages of anal disease. Differences in methylation levels were assessed using the Kruskal-Wallis test. Area under the curve was determined using logistic regression analysis. Detection rates were calculated at predefined specificities for the cross-sectional and an additional longitudinal series of 23 HGAIN biopsies preceding anal cancer (i.e., progressive HGAIN).

For both devices analytical quality criteria were met. *ASCL1* and *ZNF582* methylation levels increased with increasing severity of disease (p < 6*10^*−8*^). Diagnostic performance for AIN3^+^ was 0.81. All cancers and virtually all progressive HGAIN were detected at 70% and 80% specificity.

In conclusion, the *ASCL1/ZNF582* methylation test (PreCursor-M AnoGYN (RUO)) was demonstrated to be highly robust and reproducible. Moreover, it had excellent diagnostic accuracy to detect AIN3^+^ and can potentially be used to guide HGAIN management.

## Highlights

• Diagnostic biomarkers for cancer risk stratification of patients with high-grade anal intraepithelial neoplasia are needed.
• Elevated *ASCL1* and *ZNF582* methylation is associated with progression to cancer.
• We developed a highly robust multiplex *ASCL1/ZNF582* methylation test to guide HGAIN management.

## Abbreviations

*ACTB*: β-actin
AIN: anal intraepithelial neoplasia
AUC: area under the curve
CI: confidence interval
Cq: quantification cycle
FFPE: formalin-fixed paraffin-embedded
HGAIN: high-grade anal intraepithelial neoplasia
HIV: human immunodeficiency virus
HRA: high-resolution anoscopy
hrHPV: high-risk human papillomavirus
LoD: limit of detection
LOOCV: leave-one-out-cross-validation
MSM: men who have sex with men
MSMLWH: men who have sex with men living with HIV
PCR: polymerase chain reaction
PLWH: people living with HIV
qMSP: quantitative methylation-specific PCR
R^2^: correlations
ROC: receiver operating characteristics
RUO: research use only
SCC: squamous cell carcinoma
WTS: whole tissue sections

## 1 Introduction

High-risk human papillomavirus (hrHPV)-induced squamous cell carcinoma (SCC) of the anus is highly prevalent in specific risk groups, including people living with HIV (PLWH), men who have sex with men (MSM), transgender women, women with a history of HPV-related genital pathology and organ transplant recipients [1]. Precursor lesions of anal cancer, anal intraepithelial neoplasia (AIN) grades 2 (AIN2) and 3 (AIN3) are classified as high-grade AIN (HGAIN), can be detected using high-resolution anoscopy (HRA). Currently most HGAIN are treated with ablative or topical treatment to reduce the risk of progression to cancer [2]. However, only a limited fraction of people with HGAIN will develop anal cancer [2], leading to a large proportion of unnecessarily treated patients. This highlights the need for biomarkers to identify HGAIN patients at high risk of developing anal cancer and thus in need of treatment and on the other hand, HGAIN patients with a low cancer risk for whom a wait and see policy could be applied.

DNA methylation analysis has been proposed as a promising tool for the detection of HGAIN with an increased cancer risk [[3], [4], [5]]. DNA methylation is an epigenetic aberration that can silence tumour suppressor genes and thereby contribute to cancer development [6]. Previously, we have validated six methylation markers on two series of anal biopsies of MSM living with HIV (MSMLWH) [3,4]. In these studies, methylation levels increased with increasing severity of anal disease and methylation markers had high accuracies for the detection of AIN3 and anal SCC. HGAIN showed a heterogeneous pattern: a subset of lesions showed low methylation levels while another subset showed a “cancer-like” methylation marker pattern. The latter lesions, displaying high methylation levels, are suggested to represent advanced HGAIN with an increased cancer risk and therefore requiring direct treatment. In support of this hypothesis, we showed that a longitudinal series of HGAIN biopsies from patients that progressed to cancer over time had consistently high methylation levels [3]. A methylation marker panel consisting of the three markers *ZNF582, ASCL1,* and *SST* detected all SCCs and had an area under the curve (AUC) of 0.90 (95% confidence interval (CI): 0.86–0.94) for the detection of AIN3 and SCC. Thus, these markers proved to be a promising tool to identify HGAIN in need of treatment, preventing overtreatment of HGAIN with a low cancer progression risk [3].

For implementation of the test in diagnostics and anal cancer screening, these markers are preferably combined in one multiplex assay. Hereto, a multiplex quantitative methylation-specific PCR (qMSP) containing two target genes (*ASCL1* and *ZNF582)* and a reference gene *(ACTB)* was developed, as described previously [7]. *SST* was not included because for technical reasons it could not be combined with the other targets in one multiplex. Since the added diagnostic value of *SST* in our previous study was minimal [3], this is unlikely to negatively impact the test performance. In this study we aimed to analytically validate the *ASCL1/ZNF582* multiplex methylation assay and to determine its diagnostic performance for the detection of AIN3^+^ in comparison with our previously published assays, both in cross-sectional and longitudinal series.

## 2 Material & methods

### 2.1 Clinical specimens and ethics

A cross-sectional series of 111 formalin-fixed paraffin-embedded (FFPE) anal tissue samples was analyzed in this study (Table 1). Ninety-six AIN biopsies (23 no dysplasia, 17 AIN1, 25 AIN2, 31 AIN3), obtained from 96 HIV-positive men in screening for anal cancer between September 2018 and December 2020, were retrieved from the Amsterdam University Medical Centers (Amsterdam UMC) pathology archive. Fifteen anal SSC biopsies, both from HIV-positive and HIV-negative men and one woman, obtained between 1999 and 2020, were retrieved from the pathology archives of the Amsterdam UMC and 5 other hospitals in the Netherlands [3]. Six of these SCC biopsies were previously used in other methylation studies [3,4]. All 111 biopsies were derived from different patients.

**Table 1:** Patient characteristics.

	n	Male, n (%)	HIV^+^, n (%)	Age, mean (SD)
No dysplasia	23	23 (100%)	23 (100%)	54.1 (9.2)
AIN1	17	17 (100%)	17 (100%)	54.2 (11.6)
AIN2	25	25 (100%)	25 (100%)	53.3 (11.4)
AIN3	31	31 (100%)	31 (100%)	52.9 (10.2)
aProgressive HGAIN	10	9 (90%)	8 (80%)	b56.1 (5.9)
Anal cancer	15	14 (93%)	11 (73%)	59.8 (9.8)

a 23 biopsies derived from 10 patients.

b Age at SCC diagnosis.

The longitudinal series consisted of 23 HGAIN biopsies of 10 patients (8 HIV-positive men, 1 HIV-negative woman, 1 HIV-negative man) who developed (suspected) anal SCC over time (Table 1). From each patient, 1 or multiple consecutive HGAIN biopsies preceding the endpoint diagnosis (i.e., histopathologically confirmed SCC or ‘highly suspicious for infiltrative growth’) were defined as progressive HGAIN and included as described previously [3]. All of them were previously used in another study [3].

We adhered to the Declaration of Helsinki and Code of Conduct for Responsible Use of Left-over Material of the Dutch Federation of Biomedical Scientific Societies. Ethical approval was obtained under reference number 17/151, 18/316 and 18/333 by the Institutional Review Board of the Amsterdam UMC, location AMC. For the longitudinal series obtained outside the Amsterdam UMC, local ethical approval was granted by the NHS Health Research Authority, United Kingdom (IRAS ID 226196), and the Ethical Committee of the University Witten/Herdecke, Germany (reference no. 166/2017).

Whole tissue sections (WTS) of FFPE tissue blocks were sectioned according to the sandwich method, as described previously [3]. For confirmation of lesion presence, the first and last sections were haematoxylin and eosin stained and histopathologically reviewed by a board-certified pathologist according to the criteria, terminology and recommendations of the Lower Anogenital Squamous Terminology (LAST) project [8]. In-between WTS were collected in sterile PCR tubes for DNA isolation.

### 2.2 hrHPV detection

Samples were tested for hrHPV as described previously [3,9,10] using either the SPF_10_ DNA enzyme immunoassay (DEIA) followed by Line Probe Assay_25_ (Labo Biomedical Products B.V., Rijswijk, The Netherlands), the GP5+/6+- PCR EIA followed by Luminex typing, or the QIAscreen HPV PCR Test (Qiagen, Hilden, Germany).

### 2.3 Methylation analysis

qMSP was developed as described previously [7]. For analytical validation serial dilution series of synthetic gblocks (IDT, Leuven, Belgium) for each amplification target, ranging from 750,000 to 0.75 copies/reaction, were tested on the ViiA 7 Real-Time PCR System (Applied Biosystems, Foster City, CA, USA) and the Rotor-Gene (Qiagen) device. Linearity, reproducibility and limit of detection (LoD) was assessed by testing 6–12 technical replicates of the gblock dilution series.

For clinical samples DNA isolation and sodium bisulphite treatment were performed as described previously [11,12]. Clinical samples were tested with both the *ASCL1/ZNF582* multiplex qMSP and the original previously published assays for *ASCL1*, *ZNF582*, and *SST* on the ViiA 7 (Thermofisher) [3]. For the amplification reaction, EpiTect MethyLight Master Mix (Qiagen) was used combined with 150–300 nM of each primer and probe, as defined by primer and probe limiting assays. Amplification and real-time measurement were performed using the following conditions; 5 min at 95 °C followed by 40 cycles of 15 s at 95 °C and 1 min at 60 °C. H_2_O was tested as negative control and gblocks (250 copies/reaction) were included as positive control and for quantification. The quantification cycle (Cq) values were measured at fixed thresholds for fluorescence. ΔΔCq ratios were calculated by comparing the target Cq values to the Cq values of *ACTB* and to that of the internal quality control calibrator (i.e., gblocks) (2^**–**ΔΔCq^ x 100) [13].

To verify DNA quality and successful bisulphite conversion of clinical samples, *ACTB* was used as a reference gene in all qMSPs. A Cq threshold of <32 for *ACTB* indicated sufficient DNA and adequate bisulphite conversion [7].

### 2.4 Data analysis

For analytical validation amplification efficiencies were calculated by E=(10^(−1/Slope)^-1) × 100% for all serial dilutions above the LoD [14]. Correlations (R^2^) of the serial dilutions above LoD were determined using Microsoft Office Excel 2016. Quality criteria were set at 90% < E < 110% and R^2^ > 0.98. The LoD was based on a 95% hit rate.

To visualize methylation levels in clinical samples per disease category, boxplots were computed from the square-root transformed ΔΔCq ratios. Differences in DNA methylation levels between the disease categories were assessed using the Kruskal–Wallis omnibus test followed by *post hoc* pairwise Wilcoxon-Mann–Whitney U-tests with Bonferroni adjustment for multiple testing. Logistic regression analyses were performed to assess the risk of AIN3, AIN3^+^ (AIN3 and anal SCC) and SCC for each sample as a predicted probability (value ranging from 0 to 1). Cases were defined as AIN3, AIN3^+^ or SCC, controls were defined as ≤AIN1. These predicted probabilities were plotted in a receiver operating characteristic (ROC) curve and performance was evaluated by the area under the curve (AUC) using the references set by Hosmer and Lemeshow (i.e., 0.7 ≤ AUC <0.8 = Acceptable discrimination; 0.8 ≤ AUC <0.9 = Excellent discrimination; AUC ≥0.9 = Outstanding discrimination) [15]. We evaluated the models by leave-one-out-cross-validation (LOOCV). Predicted probabilities were calculated, based on the AIN3 versus ≤AIN1 model, for all disease states and detection rates were determined corresponding to predefined specificities of 70 and 80%. Both AUC and detection rates of the *ASCL1/ZNF582* multiplex assay were compared with that of the formerly validated three-marker panel *(ASCL1, ZNF582, SST*) using the threshold as set by van der Zee et al. [3].

Statistical analyses were performed with R Statistical Software (version 4.0.3) in RStudio, using the following R-packages: *ggplot2*, *ggpubr*, *pROC*.

## 3 Results

### 3.1 Analytical validation of the *ASCL1/ZNF582* multiplex assay

The 95% LoD was 7.5 copies/reaction for *ZNF582* and *ACTB* on both devices and for *ASCL1* on the Rotor-Gene. *ASCL1* had a LoD of 2.5 copies/reaction on the ViiA 7. Hence, PCR efficiencies and correlations were calculated for 25 copies/reaction (i.e., >LoD).

**Fig. 1:**
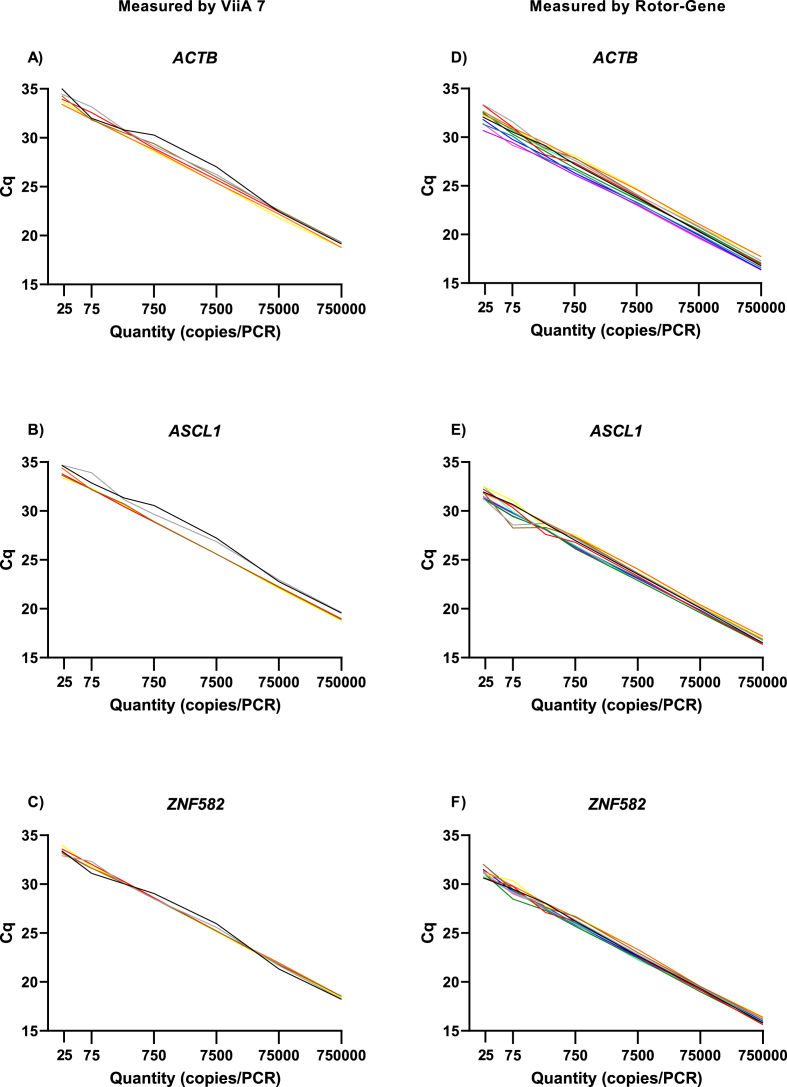
**Reproducibility of qMSPs’ per device.** Standard quantification curves obtained from 6 serial dilutions of *ACTB*, *ASCL1*, and *ZNF582* gblocks tested on the ViiA 7 (A–C), and 12 serial dilutions of *ACTB*, *ASCL1*, and *ZNF582* gblocks tested on the Rotor-Gene device (D–F). Standard quantification curves were given for dilutions above the limit of detection (i.e., ≥ 25 copies).

Testing of 6 independent serial dilution series of gblocks for each target (*ASCL1*, *ZNF582* and *ACTB)* on the ViiA 7 showed excellent correlations (R^2^ ≥ 0.986) and PCR efficiencies (range 95–103). The average R^2^ was ≥0.997 and average efficiency ranged between 99 and 100 (Table S1). Using the Rotor-Gene device 12 serial dilution series were tested and R^2^ was ≥0.979 for all targets with efficiencies ranging between 91 and 107. The average R^2^ was ≥0.996 and efficiency ranged between 98 and 99. Standard curves of independent replicates on each device are shown in Fig. 1.

### 3.2 Performance of the *ASCL1/ZNF582* multiplex assay in a cross-sectional series

**Fig. 2:**
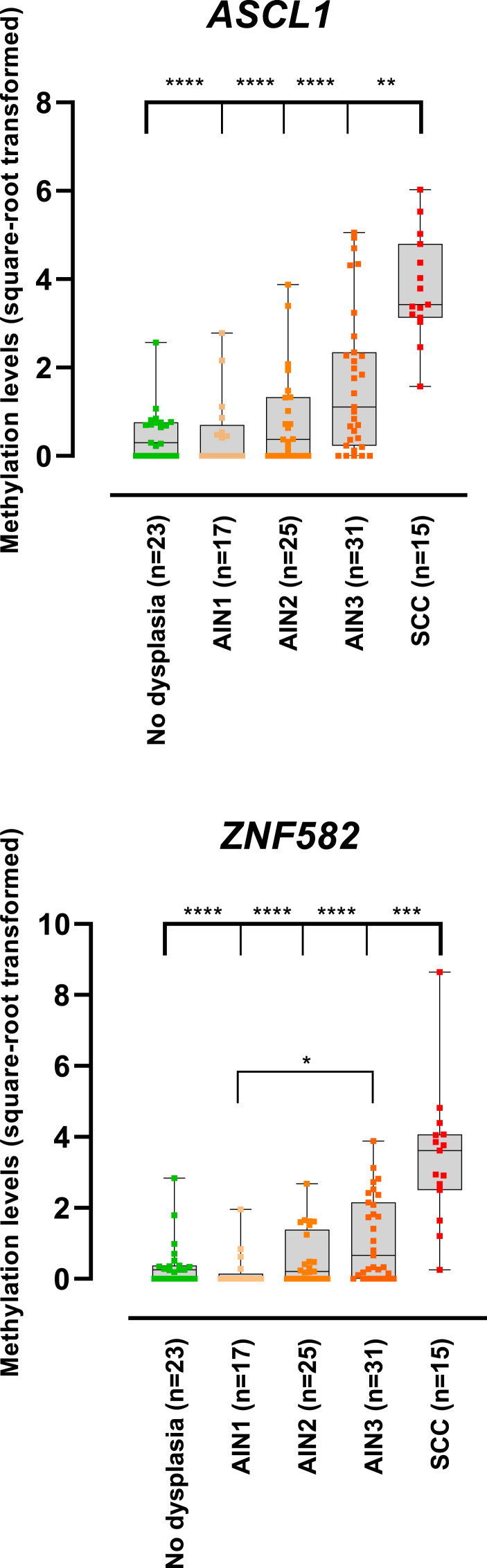
**Performance of *ASCL1/ZNF582* multiplex assay.** DNA methylation levels relative to the reference gene *ACTB* (square-root transformed ΔΔCq ratios) were plotted against the different histological outcomes of patients for 2 methylation markers: *ASCL1* and *ZNF582*. Statistical difference was assessed by the Mann-Whitney *U* test corrected for multiple testing and reported for SCC versus other histological categories (both markers) and for AIN3 versus AIN1 (*ZNF582*). Black solid line represents the median, boxplot represents the 25th (Q1) and 75th (Q3) percentile. Whiskers range from the minimum to the maximum value. * = *p* < 0.05; ** = *p* < 0.01; *** = *p* < 0.001; **** = *p* < 0.0001. Abbreviations: AIN1–AIN3, anal intraepithelial neoplasia (grades 1–3); SCC, squamous cell carcinoma.

Analysis of 111 anal tissue specimens covering all stages of disease showed that methylation levels of both *ASCL1* and *ZNF582* differed significantly between histological categories (p < 6*10^*−8*^; Fig. 2). Methylation levels were significantly higher in anal SCC as compared to AIN and normal biopsies.

**Fig. 3:**
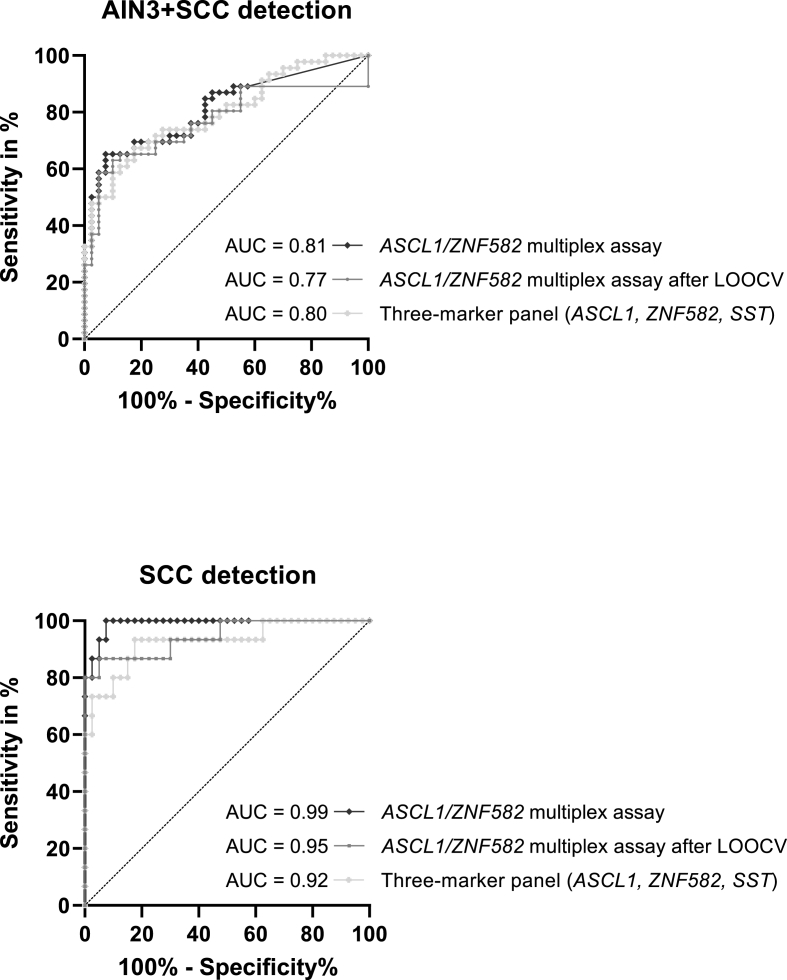
**Diagnostic performance of the different methylation assays for AIN3**^**+**^**(AIN3andSCC) and for SCC detection.** Visualized with receiver operating characteristic curves for the *ASCL1/ZNF582* multiplex assay and for the model corresponding with the formerly validated three-marker panel *(ASCL1, ZNF582, SST*) as defined by van der Zee et al. [3]. Controls = no dysplasia and AIN1 biopsies. Abbreviations: AUC: Area under the curve; SCC: squamous cell carcinoma.

The ability of the *ASCL1/ZNF582* multiplex assay to detect AIN3 was acceptable (AUC of 0.73), to detect AIN3^+^ it was excellent (AUC of 0.81), and to detect anal SCC it was outstanding (AUC of 0.99). After LOOCV, AUCs of 0.65, 0.77 and 0.95 were obtained for AIN3, AIN3^+^ and anal SSC, respectively (Fig. 3). A virtually equal performance was obtained upon analysis of the same samples with the original qMSP and validated three-marker panel using the threshold as set by van der Zee et al. [3], with AUCs for AIN3, AIN3^+^ and SCC detection of 0.74, 0.80 and 0.92, respectively.

When applying the model, based on AIN3 versus ≤AIN1, 48.2% of HGAIN and 30.0% of ≤AIN1 were methylation positive at a predefined 70% specificity. At a predefined 80% specificity, 41.1% of HGAIN and 20.0% of ≤AIN1 were methylation positive. These numbers were 53.6% (i.e., HGAIN) and 32.5% (i.e., ≤AIN1) when using the originally validated assay. When applying the model and corresponding thresholds to the cancers, all were methylation positive. With hrHPV testing, 15.4% of ≤AIN1, 83.9% of HGAIN and all cancers were positive (Table 2).

**Table 2:** Methylation positive detection rate by the formerly validated three-marker panel *(ASCL1, ZNF582, SST*) and the newly developed *ASCL1/ZNF582* multiplex assay.

	No dysplasia, n (%)	AIN1, n (%)	AIN2, n (%)	AIN3, n (%)	SCC, n (%)
hrHPV testing	2/23 (8.7)	d4/16 (25.0)	19/25 (76.0)	28/31 (90.3)	15/15 (100.0)
aThree-marker panel *(ASCL1, ZNF582, SST*)	8/23 (34.8)	5/17 (29.4)	10/25 (40.0)	20/31 (64.5)	14/15 (93.3)
b*ASCL1/ZNF582* multiplex assay: 70% specificity	8/23 (34.8)	4/17 (23.5)	9/25 (36.0)	18/31 (58.1)	15/15 (100.0)
c*ASCL1/ZNF582* multiplex assay: 80% specificity	4/23 (17.4)	4/17 (23.5)	7/25 (28.0)	16/31 (51.6)	15/15 (100.0)

a Threshold was set as defined by van der Zee et al. [3].

b Threshold was set corresponding to a predefined specificity of 80% with cases defined as AIN3 and controls as ≤AIN1.

c Threshold was set corresponding to a predefined specificity of 70% with cases defined as AIN3 and controls as ≤AIN1.

d For 1 of the AIN1 biopsies no hrHPV testing was performed.

### 3.3 Association of *ASCL1/ZNF582* positivity with progression to cancer

In the longitudinal series, all progressive HGAIN (i.e., HGAIN preceding SCC) consistently showed high methylation levels, comparable to the highest levels in AIN3 and cancers in the cross-sectional series (Supplementary Figure 1). All but one of these biopsies scored methylation positive according to the model using the thresholds corresponding to a given specificity of 70 and 80%. The single biopsy scoring methylation negative was just below the 70% specificity threshold. All progressive HGAIN were hrHPV positive.

## 4 Discussion

We tested the analytical validity and diagnostic performance of the *ASCL1/ZNF582* multiplex methylation assay, currently commercialized as the PreCursor-M AnoGYN research use only (RUO) test. The *ASCL1/ZNF582* multiplex was highly reproducible and showed good linearity on both the ViiA 7 and the Rotor-Gene device, with an average R^2^ of 0.997 and an average efficiency of 98, respectively. Also an analytical sensitivity of 7.5–2.5 copies/reaction was reached for the different markers. The assay thereby meets predefined quality criteria for use in diagnostics [7].In anal biopsies methylation levels for both *ASCL1* and *ZNF582* increased with increasing severity of the disease and were highest in SCC. The diagnostic performance of the *ASCL1/ZNF582* multiplex for the detection of AIN3 and SCC (i.e., 0.81) was equal to that of the formerly validated three-marker panel (i.e., 0.80), both of which were tested on the same sample series. Using the *ASCL1/ZNF582* multiplex methylation assay with a threshold corresponding to predefined specificities of 70% and 80%, 51.8 and 58.9% of HGAIN biopsies were methylation negative. Thus, 51.8–58.9% fewer patients would have been treated while still detecting all cancers and virtually all progressive HGAIN. Using hrHPV testing, only 16.1% of HGAIN biopsies were negative indicating that risk stratification by hrHPV is of limited value*.*

The *ASCL1/ZNF582* multiplex demonstrated an almost equal diagnostic performance as the previously validated three-marker panel consisting of *ASCL1*, *ZNF582* and *SST* tested on the same sample series. Nonetheless, the AUC for AIN3^+^ was lower in this study (AUC: 0.81 and 0.80) than in previous studies (AUC: 0.90 and 0.89) [3,4]. This can be explained by the differences in populations, as a) the previous studies used a cohort of patients without a history of HGAIN screening or treatment, b) in the previous cohort combination antiretroviral therapy was initiated at a later stage according to previous guidelines, and c) a large part of the population of the current study received regular screening and treatment of HGAIN when needed. Therefore, fewer cases of advanced HGAIN can be expected. With fewer advanced cases, the diagnostic accuracy of any test will automatically decrease. Another reason for the difference in the accuracy might be the smaller sample size of current study, which is therefore more prone to variability.

The ability of the test to detect those lesions at highest risk of progressing to cancer is supported by virtually all progressive HGAIN and all anal cancers testing methylation positive at a specificity of 70% and 80% for AIN3 detection. The cancers tested in this study also included HIV-negative cases and women, indicating that the test can have a wider application than MSMLWH. Concordantly, in our previous study a similar performance of methylation testing was demonstrated for anal biopsies of PLWH in general and people living without HIV [9]. Nonetheless, extension to risk groups other than MSMLWH requires further validation.

As a test with a continuous outcome, the *ASCL1/ZNF582* multiplex has the advantage of flexible use of clinical action thresholds that can be modified according to the setting it is implemented in. A proposed clinical action threshold would be to distinguish advanced HGAIN with an increased chance of progression to cancer and in need of immediate treatment, from HGAIN with a low cancer risk for which a wait-and-see policy may be implemented. Based on the samples included in this analysis a specificity just below 70% would enable the detection of all progressive HGAIN. Nonetheless, for use in clinical practice, it is essential to have a careful discussion with an expert panel (e.g. using a Delphi method) to determine the required specifications. Furthermore, clinical guidelines need to be established, such as screening intervals and long-term safety after a negative test.

The next step towards implementation of the *ASCL1/ZNF582* multiplex is clinical validation in a prospective study. Currently, we are performing a prospective cohort of MSMLWH with HGAIN that are actively monitored and thus receive no treatment for two years (NTR number = NL9664) [16]. Here, the *ASCL1/ZNF582* multiplex will be tested on baseline biopsies and we will evaluate whether regression of HGAIN can be predicted by a negative methylation test.

A strength of this study is the direct comparison of the *ASCL1/ZNF582* multiplex with the originally validated three-marker panel and with hrHPV testing in a screening setting. Furthermore, we compared two different PCR devices of two manufacturers, which yielded nearly equal results, suggesting high reproducibility. A limitation of this study is the relatively small sample size (n = 134) compared to previous studies (n = 148 and n = 345) [3,4], although it was sufficient for the purpose of the present study aiming to train the multiplex test and to compare its performance to the formerly validated assays.

Concluding, the newly developed *ASCL1/ZNF582* multiplex qMSP is highly robust and reproducible and has a good analytical sensitivity in different PCR devices. The *ASCL1/ZNF582* methylation test had a good and at least equal diagnostic performance as our earlier designed and validated assays. It has great potential in reducing overtreatment of HGAIN by accurately distinguishing between those lesions that require treatment and those that can be safely monitored without intervention.

## Funding

FDGL was supported by an AMC PhD Scholarship, and KR by a grant from Stichting Life Sciences & Health. The source of funding did not have any influence on the design of the study, collection, analysis, interpretation of the data, writing of the manuscript, or the decision to submit the article for publication.

## CRediT authorship contribution statement

**Kirsten Rozemeijer:** Data curation, Formal analysis, Investigation, Methodology, Visualization, Writing – original draft, Writing – review & editing. **Fernando Dias Gonçalves Lima:** Methodology, Resources, Validation, Writing – original draft, Writing – review & editing. **Timo J. ter Braak:** Investigation, Resources, Writing – review & editing. **Albertus T. Hesselink:** Writing – review & editing. **Jan M. Prins:** Resources, Writing – review & editing. **Henry J.C. de Vries:** Resources, Writing – review & editing. **Renske D.M. Steenbergen:** Conceptualization, Methodology, Resources, Supervision, Writing – review & editing.

## Declaration of competing interest

The authors declare the following financial interests/personal relationships which may be considered as potential competing interests: RDMS is a minority stockholder of Self-screen B.V., a spin-off company of VUmc, which owns patents on methylation markers and HPV detection. HJCdV received financial compensation or goods for research from Medigene, Gilead, and MSD; financial compensation for presentations from Abbott and Janssen; and financial compensation for advice to Medigene and Novartis. All other authors report no potential conflicts.

## Data Availability

Data will be made available on request.
