# Supplementary material for: Analytical validation and diagnostic performance of the ASCL1/ZNF582 methylation test for detection of high-grade anal intraepithelial neoplasia and anal cancer
Source: Tumour Virus Res. 2023 Dec 30;17:200275. doi: 10.1016/j.tvr.2023.200275 (PMC10821616; doi:10.1016/j.tvr.2023.200275)
Supplement: Multimedia component 1 [file mmc1.docx]

**Supplementary Figure 1. Performance of *ASCL1/ZNF582* multiplex assay including progressive HGAIN**

DNA methylation levels relative to the reference gene *ACTB* (square-root transformed ΔΔCq ratios) were plotted against the different histological outcomes of patients for 2 methylation markers: *ASCL1* and *ZNF582*. Statistical difference was assessed by the Mann-Whitney *U* test corrected for multiple testing and reported for SCC versus other histological categories (both markers) and for AIN3 versus AIN1 (*ZNF582*). Black solid line represents the median, boxplot represents the 25^th^ (Q1) and 75^th^ (Q3) percentile. Whiskers range from the minimum to the maximum value.

* = *p* < 0.05; ** = *p* < 0.01; *** = *p* < 0.001; **** = *p* < 0.0001. Abbreviations: AIN1–AIN3, anal intraepithelial neoplasia (grades 1–3); SCC, squamous cell carcinoma.
