# Supplementary material for: Analytical validation and diagnostic performance of the ASCL1/ZNF582 methylation test for detection of high-grade anal intraepithelial neoplasia and anal cancer
Source: Tumour Virus Res. 2023 Dec 30;17:200275. doi: 10.1016/j.tvr.2023.200275 (PMC10821616; doi:10.1016/j.tvr.2023.200275)
Supplement: Multimedia component 2 [file mmc2.pdf]

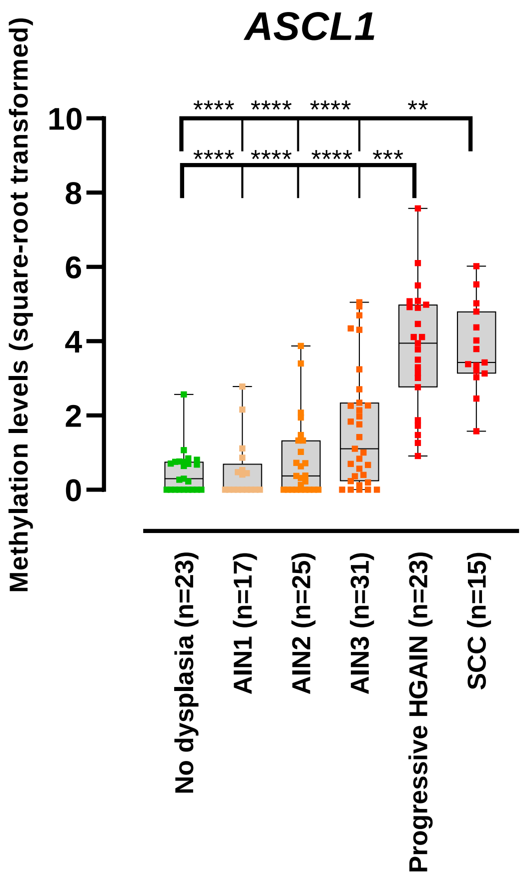

ASCL1
Methylation levels (square-root transformed)
10
8
6
4
2
0
****
****
****
**
****
****
****
***
No dysplasia (n=23)
AIN1 (n=17)
AIN2 (n=25)
AIN3 (n=31)
Progressive HGAIN (n=23)
SCC (n=15)


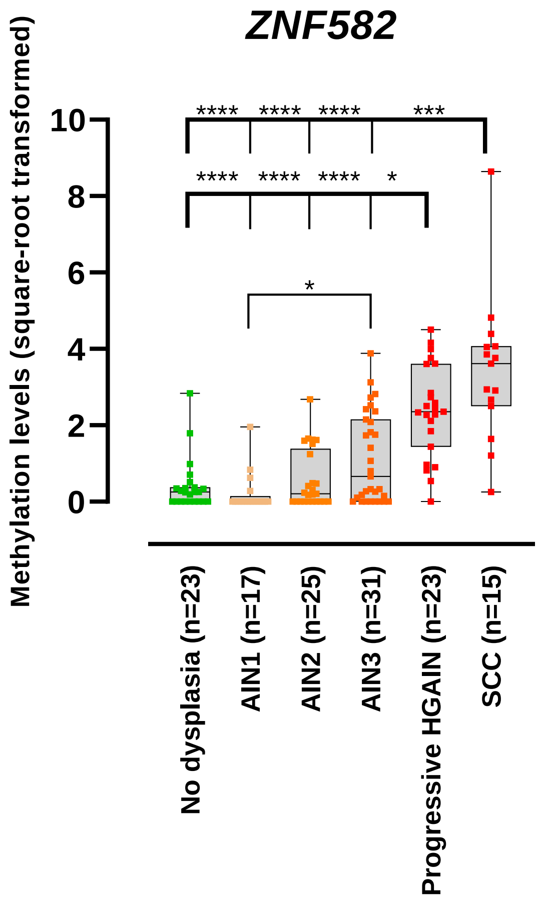

ZNF582
Methylation levels (square-root transformed)
10
8
6
4
2
0
****
****
****
***
****
****
****
*
*
No dysplasia (n=23)
AIN1 (n=17)
AIN2 (n=25)
AIN3 (n=31)
Progressive HGAIN (n=23)
SCC (n=15)
