# Supplementary material for: Analytical validation and diagnostic performance of the ASCL1/ZNF582 methylation test for detection of high-grade anal intraepithelial neoplasia and anal cancer
Source: Tumour Virus Res. 2023 Dec 30;17:200275. doi: 10.1016/j.tvr.2023.200275 (PMC10821616; doi:10.1016/j.tvr.2023.200275)
Supplement: Multimedia component 3 [file mmc3.docx]

**Supplementary Table 1. Reproducibility of the *ASCL1/ZNF582* multiplex assay**

|  |  | ViiA 7 device | | | Rotor-Gene device | | |
| --- | --- | --- | --- | --- | --- | --- | --- |
|  |  | ***ACTB*** | ***ASCL1*** | ***ZNF582*** | ***ACTB*** | ***ASCL1*** | ***ZNF582*** |
| Run 1 | E | 97 | 98 | 99 | 95 | 94 | 98 |
|  | R^2^ | 0.986 | 0.993 | 0.990 | 0.999 | 1.000 | 0.998 |
| Run 2 | E | 96 | 96 | 99 | 91 | 107 | 99 |
|  | R^2^ | 0.998 | 0.996 | 0.996 | 0.998 | 0.986 | 0.999 |
| Run 3 | E | 103 | 100 | 101 | 98 | 106 | 96 |
|  | R^2^ | 0.997 | 1.000 | 1.000 | 0.999 | 0.979 | 0.996 |
| Run 4 | E | 101 | 100 | 98 | 91 | 94 | 97 |
|  | R^2^ | 0.999 | 1.000 | 1.000 | 0.993 | 0.996 | 0.997 |
| Run 5 | E | 102 | 97 | 102 | 100 | 100 | 98 |
|  | R^2^ | 1.000 | 0.999 | 0.999 | 1.000 | 0.999 | 0.999 |
| Run 6 | E | 99 | 100 | 95 | 101 | 94 | 99 |
|  | R^2^ | 1.000 | 0.999 | 0.999 | 0.997 | 0.997 | 0.996 |
| ^*^Run 7 | E | N.a. | N.a. | N.a. | 98 | 100 | 99 |
|  | R^2^ | N.a. | N.a. | N.a. | 0.998 | 0.999 | 0.997 |
| ^*^Run 8 | E | N.a. | N.a. | N.a. | 95 | 92 | 99 |
|  | R^2^ | N.a. | N.a. | N.a. | 0.999 | 0.996 | 0.999 |
| ^*^Run 9 | E | N.a. | N.a. | N.a. | 101 | 100 | 94 |
|  | R^2^ | N.a. | N.a. | N.a. | 0.999 | 1.000 | 0.996 |
| ^*^Run 10 | E | N.a. | N.a. | N.a. | 98 | 100 | 98 |
|  | R^2^ | N.a. | N.a. | N.a. | 0.999 | 1.000 | 0.998 |
| ^*^Run 11 | E | N.a. | N.a. | N.a. | 105 | 101 | 99 |
|  | R^2^ | N.a. | N.a. | N.a. | 0.999 | 1.000 | 1.000 |
| ^*^Run 12 | E | N.a. | N.a. | N.a. | 101 | 98 | 98 |
|  | R^2^ | N.a. | N.a. | N.a. | 0.999 | 1.000 | 0.999 |
| On average | E | 100 | 99 | 99 | 98 | 99 | 98 |
|  | R^2^ | 0.997 | 0.998 | 0.997 | 0.998 | 0.996 | 0.998 |

^*^6 Dilution runs were performed with the ViiA 7 as compared to 12 with the Rotor-Gene device.

Abbreviations: E: Amplification efficiencies; N.a.: Not applicable; R^2^: Correlation coefficient.
